# ESR Essentials: thyroid imaging—practice recommendations by the European Society of Head and Neck Radiology

**DOI:** 10.1007/s00330-025-12101-2

**Published:** 2025-11-19

**Authors:** Edith Vassallo, Anne Péporté, Andrew McQueen, Minerva Becker, Jussi Hirvonen

**Affiliations:** 1 Medical Imaging Department, Mater Dei Hospital, Imsida, Malta; 2 Department of Radiology, Cantonal Hospital, Frauenfeld, Switzerland; 3 Institute of Diagnostic and Interventional Radiology, University Zurich, Zurich, Switzerland; 4 Department of Radiology, Freeman Hospital, Newcastle upon Tyne Hospitals NHS Foundation Trust, Newcastle, UK; 5 Division of Radiology, Diagnostic Department, Geneva University Hospitals, Geneva, Switzerland; 6 Faculty of Medicine, University of Geneva, Geneva, Switzerland; 7 Department of Radiology, University of Turku and Turku University Hospital, Turku, Finland; 8 Department of Radiology Tampere University, Faculty of Medicine and Health Technology, Tampere University Hospital, Tampere, Finland

**Keywords:** Thyroid, Nodule, TI-RADS, Fine-needle aspiration, Multidisciplinary

## Abstract

**Abstract:**

Thyroid nodules are frequently encountered at imaging, yet most are benign and do not require intervention. The clinical challenge lies in distinguishing nodules that warrant further investigation from those that do not, to avoid unnecessary biopsies, anxiety, and overtreatment. Ultrasound (US) is the primary imaging modality for thyroid nodule evaluation, supported by structured risk stratification systems such as ACR TI-RADS and EU-TIRADS, which incorporate specific sonographic features and size thresholds to guide clinical decision-making. Nodules without high-risk features can be safely monitored or ignored, especially in asymptomatic patients. Conversely, suspicious characteristics (e.g. irregular margins, microcalcifications, or marked hypoechogenicity) should prompt further assessment, including fine-needle aspiration (FNA). Diffuse thyroid disorders, including Hashimoto’s thyroiditis and Graves’ disease, are best assessed using US and thyroid function tests. In thyroid cancer, a multidisciplinary team approach involving radiologists, endocrinologists, pathologists, and nuclear medicine specialists is essential for optimal patient care. To implement these recommendations, radiologists should adopt standardised US reporting systems, apply evidence-based criteria for further workup, and collaborate closely with referring clinicians. This approach ensures accurate diagnosis, reduces unnecessary procedures, and aligns radiological practice with current guidelines to support high-value, patient-centred care.

**Key Points:**

- *Ultrasound (US) is the gold standard imaging modality for evaluating thyroid pathology*.
- *Implementation of the EU-TIRADS and ACR-TIRADS constitutes a critical part of the work-up of thyroid nodules and is essential for their effective management*.
- *Interdisciplinary discussion with all specialists concerned is the most effective way of ensuring that the best possible management strategy is implemented in thyroid cancers*.

## Key recommendations

- Reserve further investigations of incidentally detected thyroid nodules (ITNs) selectively by evaluating only ITNs with high-risk imaging features or clinical indicators, and avoid routine workup of nodules lacking suspicious sonographic characteristics (level of evidence: high).
- Employ validated standardised risk stratification scoring (RSS) frameworks such as ACR TI-RADS or EU-TIRADS to ensure consistent, evidence-based evaluation and appropriate use of fine-needle aspiration (FNA) (level of evidence: high).
- Promote multimodal, multidisciplinary management of thyroid cancer (TC) involving radiologists, endocrinologists, surgeons, and nuclear medicine specialists to guide individualised management based on tumour biology and patient-specific prognostic factors (level of evidence: high).

## Introduction

Thyroid disorders are common, with imaging playing a critical role in addressing the challenges they pose. As ultrasound (US), CT, and MRI scans become routine, radiologists frequently encounter thyroid findings, often unexpectedly, ranging from incidental nodules to diffuse gland abnormalities and disseminated cancer. These discoveries demand informed decisions to guide patient care, yet the nuances of thyroid imaging can challenge even experienced radiologists. Incidental nodules raise questions about malignancy risk, while diffuse diseases require careful differentiation based on subtle imaging cues. The advent of risk stratification systems (RSS) based on US features has brought structure to nodule evaluation, but applying it consistently across practice settings remains a hurdle. For TC, imaging is pivotal, dictating staging, treatment, and surveillance, yet guidelines evolve rapidly, leaving gaps in practical application. Radiologists need clear, evidence-based strategies to navigate these scenarios effectively, ensuring accurate diagnoses and avoiding unnecessary interventions. This article provides practice recommendations for imaging choices and diagnostic considerations, when evaluating thyroid diseases.

## Incidental ITNs

Asymptomatic ITNs are often discovered incidentally during imaging. The vast majority of Incidental ITNs are benign, whilst malignant ITNs are typically well-differentiated thyroid cancers (DTC), which tend to follow an indolent course and carry an excellent prognosis. The radiologist plays a critical role when reporting ITNs, as over-detection and overemphasis of disease may ultimately cause more harm than benefit.

### The scale of the challenge

**Fig. 1:**
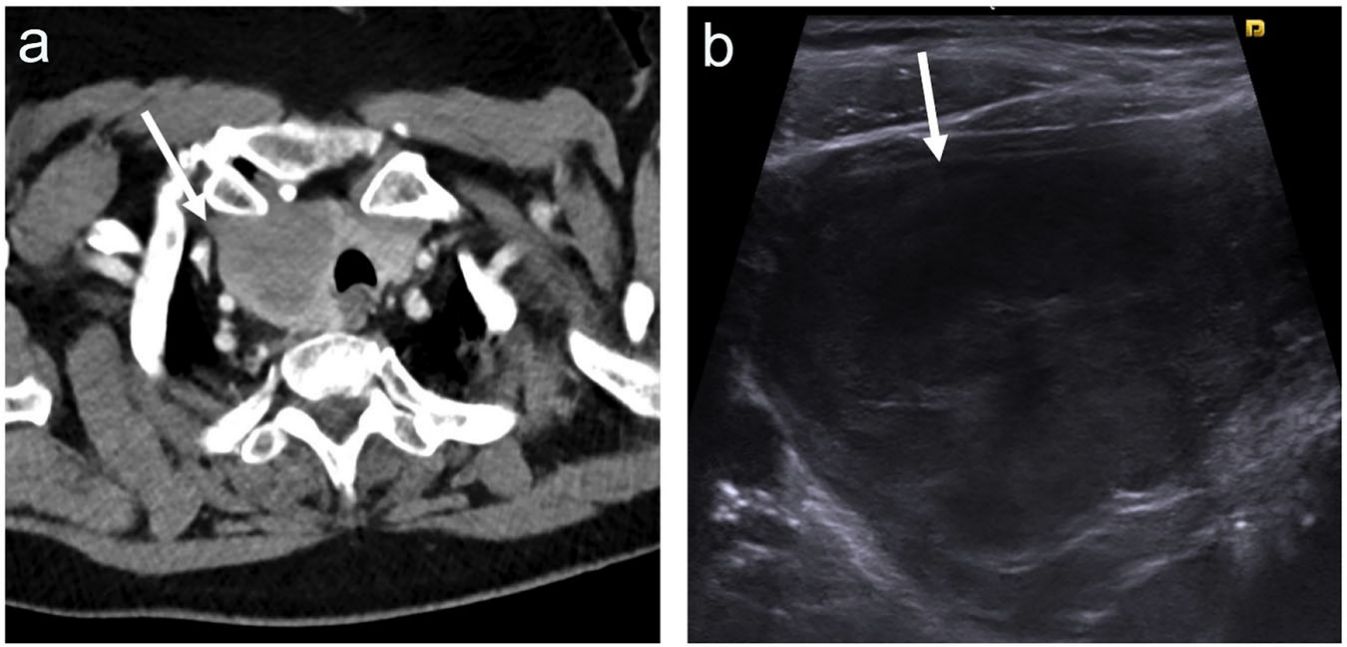
Incidental right thyroid lobe lesion with suspicion of extrathyroidal extension (ETE) on CT (arrow in (**a**)). On ultrasound RSS (**b**), the right thyroid lobe is replaced by a solid hypoechoic mass that bulges into the strap muscle (arrow), suggestive of malignancy. Core needle biopsy demonstrated diffuse large B-cell lymphoma

**Fig. 2:**
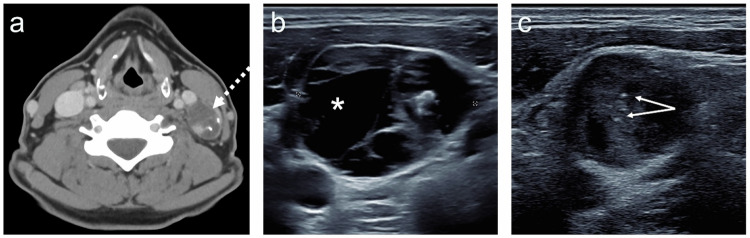
**a** Incidental CT finding of an abnormal left cervical (level 3) lymph node (dotted arrow) containing calcification and cystic spaces in a patient with small ITNs. **b**, **c** US demonstrated mixed isoechoic solid and anechoic cystic consistency (asterisk) with punctate echogenic foci consistent with microcalcification (arrows). FNA from the level 3 lymph node demonstrated papillary thyroid carcinoma with surgical confirmation (pT1 8 mm micro papillary thyroid carcinoma pN1b)

ITNs are commonly detected during cross-sectional imaging of the lower neck, with a reported prevalence of 16–25% in adult populations [1]. Contrast-enhanced CT is the leading modality for ITN identification, particularly in chest and neuroradiology studies. Detection rates increase with age and are higher in women [2]. Imaging findings such as locoregional invasion (extrathyroidal extension) or cystic/calcified lymphadenopathy raise suspicion for TC but are uncommon, especially in asymptomatic patients (Figs. 1 and 2). Most ITNs lack specific features, and CT/MRI perform poorly in characterising nodule nature, earning the label: ‘easily identified, poorly characterised’.

Due to its superior spatial resolution, neck US has higher sensitivity and specificity in comparison to other imaging modalities. US enables  risk stratification scoring (RSS) for  thyroid nodules, and several RSS schemes have been published so far. Proper RSS classification addresses interobserver variability and reduces unnecessary investigations, e.g. FNA by distinguishing benign from suspicious nodules [3].

Hybrid imaging, especially ^18^F-fluorodeoxyglucose (FDG) PET-CT, identifies focal thyroid uptake in 1-2.5% of studies, making it the second most frequent site of PET-CT incidentalomas after the colon [4]. A meta-analysis of over 147,000 PET-CT examinations showed a pooled malignancy risk of 34.6% in FDG-avid ITNs, with most cancers being small papillary thyroid carcinomas (PTCs). Whilst US and FNA are standard recommendations for PET-positive ITNs, the clinical context is crucial. In oncologic patients with ITNs detected on PET-CT, median survival from the primary malignancy is only 20 months, with > 99% of deaths unrelated to thyroid disease [5]. Table 1 summarises key features of ITNs based on the experiences from countries with the highest rates of ITN investigation [1, 6].

**Table 1:** Key features of incidental ITNs

Feature	Incidental thyroid nodule
Modality of detection	CT » MRI » PET-CT
Size	< 20 mm in 49% of cases
Risk of malignancy	1.6–2.8% (CT, MRI, US) 30.8–34.6% ([^18^F]FDG PET-CT)
Type of malignancy	Papillary thyroid carcinoma > 90%
10-year survival (< 3 cm malignant ITN)	99.4%
Risk of an inconclusive FNA result	> 20%

### What we know about ITNs

**Fig. 3:**
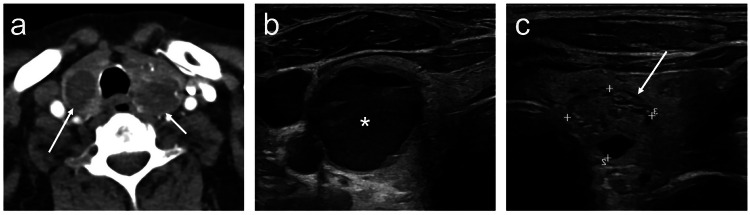
Bilateral ITNs identified on CT (carotid angiogram) (**a**) in an asymptomatic adult. **a** CT does not characterise the  thyroid nodules further. **b**, **c** On high-resolution US, the nodules’ features of cystic consistency (asterisk), well-defined margins, and spongiform  morphology (arrow) result in a benign grading and no FNA

In healthcare systems where ITNs commonly lead to further investigation, US-based RSS is crucial for FNA decision (Fig. 3). When FNA is performed, benign and malignant cytology results carry high negative and positive predictive values, respectively [7]; however, > 20% of FNA cases may yield non-diagnostic or indeterminate cytology results. In this scenario, the nature of the nodule and the management plan remain uncertain, leaving options of repeat FNA, US surveillance, or diagnostic thyroid surgery. This diagnostic process is often referred to as a ‘cascade of care’, whereby asymptomatic individuals undergo multiple diagnostic steps to reach a definitive diagnosis and to determine management.

### Risk and harm associated with ITNs

The detection of incidental TCs, particularly through imaging, often leads to an overestimation of clinical risk by both patients and radiologists. There exists a widespread concern that missing an early TC diagnosis could result in delayed treatment and poorer outcomes. However, the majority of malignant ITNs represent small DTCs with indolent behaviour and excellent long-term prognosis. This misconception is exemplified by the South Korean experience, where widespread thyroid US screening has led to a 15-fold increase in TC diagnosis (mostly DTCs < 1 cm) without a corresponding change in mortality [8]. This phenomenon, now widely recognised as overdiagnosis, has similarly been reported in other countries where imaging surveillance is prevalent.

A key epidemiological concept in understanding ITNs is length bias (i.e. an apparent increase in survival in the screening scenario due to detecting disease earlier than it would otherwise have been detected). Indolent DTCs, due to their slow growth, are far more likely to be detected incidentally during routine imaging, whereas aggressive TCs, which grow rapidly and present symptomatically, are rarely detected incidentally. Thus, while ITNs may be numerous, they seldom represent aggressive malignancy. Additionally, investigating ITNs in older populations has limited benefit, as 80% of ITN patients die of non-thyroid causes within 13 years, highlighting the minimal clinical impact of many ITNs [9]. Aggressive TCs or metastases are rare (1%) [10].

The physical harm from ITN diagnosis primarily relates to complications from surgery (scarring, lifelong thyroxine replacement, vocal cord paralysis). These outcomes are well recognised and routinely discussed during patient consent.

For patients with ITN investigations, studies have shown anxiety (‘fear of cancer’) even when the US RSS is benign and FNA is not performed [10]. For those diagnosed with TC, persistent anxiety, depression, and fatigue, often comparable to or exceeding that seen in survivors of more aggressive cancers, have been reported [11]. Notably, these effects persist despite a generally excellent prognosis and normal life expectancy. Even in cases managed conservatively with active surveillance, particularly among younger patients, elevated anxiety levels are common, questioning the mental health impact of current surveillance guidelines.

From an economic perspective, ITN overdiagnosis imposes a substantial burden. In the United States alone (where most TC diagnoses arise from ITN investigations), the cost of diagnosing, treating, and monitoring DTC is projected to reach $3.5 billion annually by 2030 [12]. In contrast, countries with lower rates of ITN investigation (e.g. Nordic countries) have avoided such cost escalations. On a personal level, more than half of patients report financial hardship following DTC diagnosis [13], and TC has been associated with a higher risk of bankruptcy than many other cancers. As patient access to imaging expands globally, the financial implications of ITN management, particularly in low- and middle-income countries, will become even more pressing.

To mitigate harm from ITNs, avoiding unnecessary thyroid imaging is a key strategy. Numerous national bodies, including the United States Preventive Services Task Force, advise against TC screening in asymptomatic individuals [14]. National guidelines for ITN reporting provide a standardised approach; however, current national recommendations differ significantly from one country to another. The American College of Radiology (ACR) advises US-based RSS for every ITN > 1.5 cm in diameter (1.0 cm if < 35 years) without malignant features, unless there is ‘limited life expectancy or co-morbidities’ [15]. Conversely, the United Kingdom National Institute for Health and Care Excellence (NICE) advises against routine RSS for ITNs due to potential harms [16].

## Risk stratification of ITNs based on sonographic features

**Fig. 4:**
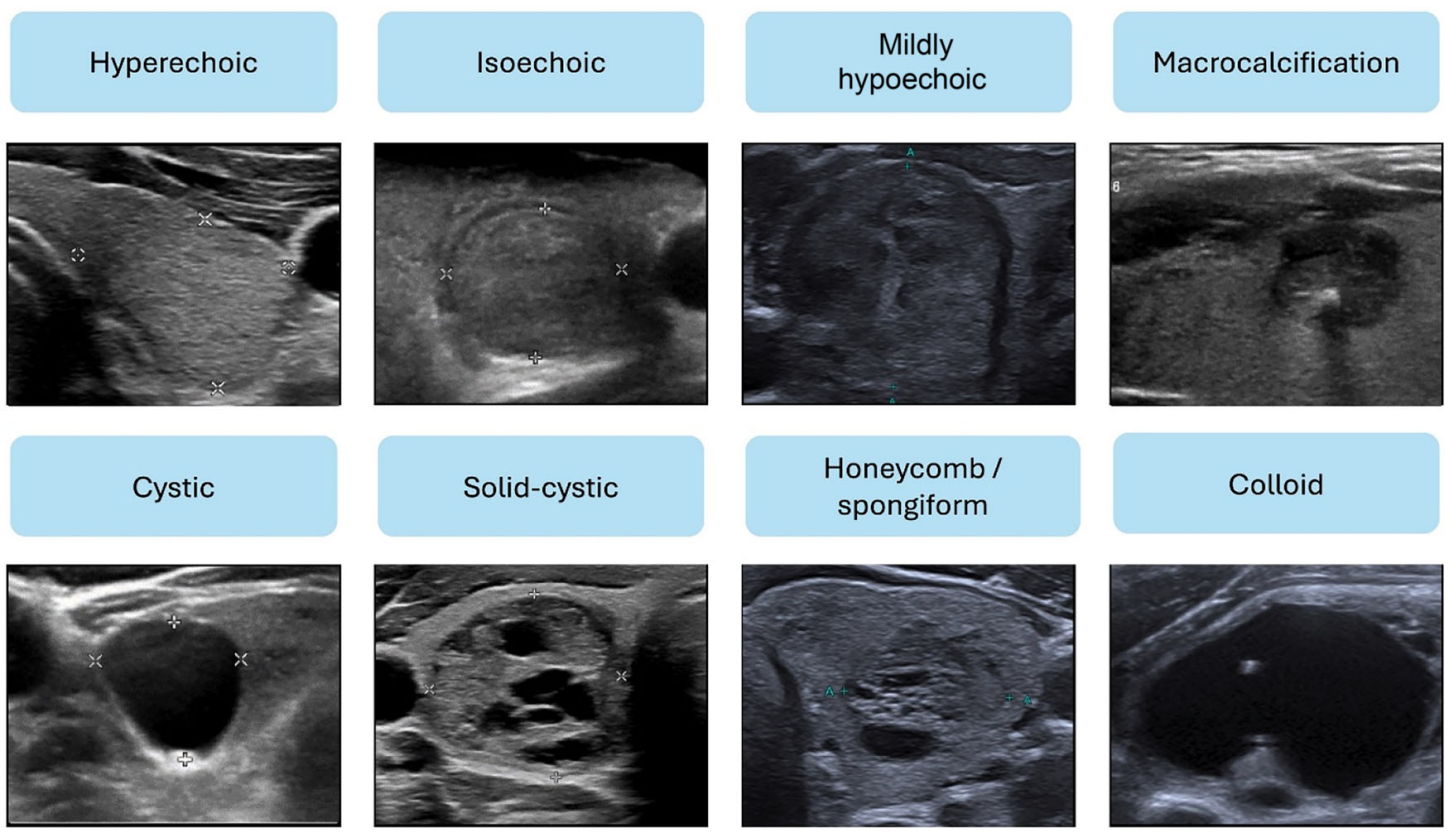
US features of ITNs based on a consensus lexicon, typically associated with benign aetiology

**Fig. 5:**
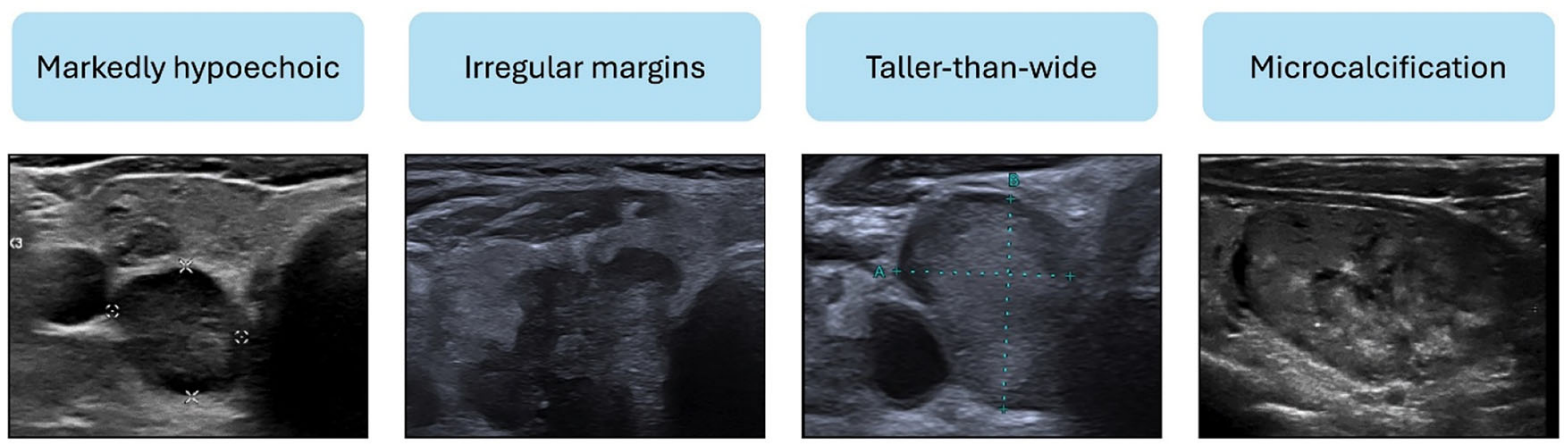
US features of ITNs suspicious for malignancy, based on a consensus lexicon

US has become the primary imaging modality for evaluating ITNs, with standardised RSS developed to differentiate benign from malignant lesions. A consensus lexicon of the relevant US features, such as composition, echogenicity, margins, and calcifications, has been published [17]. Cystic or spongiform morphology typically suggests benign pathology (Fig. 4). Notably, nodule size is an unreliable predictor of malignancy. Conversely, the features with the highest predictive value for malignancy are taller-than-wide morphology, microcalcifications, irregular margins, and marked hypoechogenicity (Fig. 5) [18].

The American Thyroid Association (ATA) guidelines [19] and the ACR Thyroid Imaging Reporting and Data System (ACR TI-RADS) introduced a point-based scoring algorithm that assigns malignancy risk based on discrete ultrasound features—composition, echogenicity, shape, margin, and echogenic foci—to categorise nodules from TR1 (benign) to TR5 (high suspicion) [19]. FNA thresholds depend on both category and nodule size: ≥ 2.5 cm (TR3), ≥ 1.5 cm (TR4), and ≥ 1.0 cm (TR5), aiming to minimise overdiagnosis while ensuring malignancies are not missed. These guidelines have been instrumental in guiding clinical decision-making and reducing unnecessary biopsies. Other RSS schemes have also been developed, the most commonly used being the European (EU-TIRADS) [3] and the Korean (K-TIRADS) [20] systems.

In Europe, the EU-TIRADS [3], which is recommended by the European Thyroid Association [3], is the predominant system. EU-TIRADS employs a pattern-based classification stratifying nodules into five categories (EU-TIRADS 1-5) based on ultrasound patterns, with FNA recommended at > 20 mm (EU-TIRADS 3), > 15 mm (EU-TIRADS 4), and > 10 mm (EU-TIRADS 5). These differences reflect EU-TIRADS’s focus on streamlined pattern recognition versus ACR TI-RADS’s quantitative precision. Direct comparisons [21] have shown that both the ACR and the EU systems effectively stratify thyroid nodule malignancy risk, with comparable overall diagnostic performance. ACR TI-RADS reduces unnecessary FNAs more effectively due to stricter criteria and higher specificity, particularly for high-risk categories, while EU-TIRADS’s simpler approach may increase FNA indications due to its sensitivity-driven, single-feature-based high-risk classification. Taken together, ACR TI-RADS and EU-TIRADS seem to be equally valid options for RSS evaluation. An International Thyroid Imaging Reporting and Data System, or I-TIRADS, is currently being developed [22].

## Diffuse thyroid disorders (DTD)

DTD such as Hashimoto’s thyroiditis and Graves’ disease, are primarily diagnosed via clinical and biochemical evaluation, with US as the gold standard imaging modality.

**Fig. 6:**
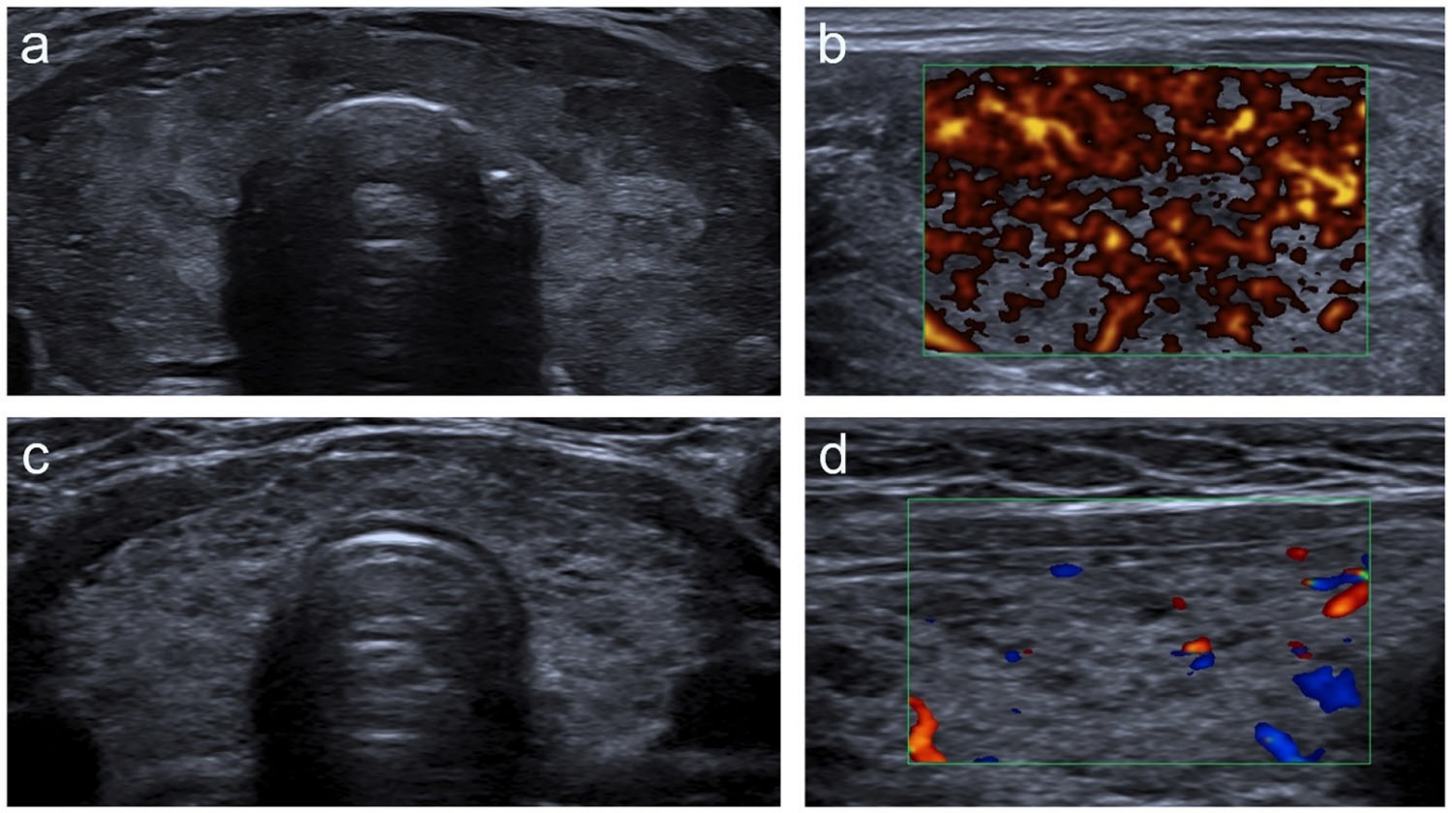
Sonographic features of diffuse thyroid disease in two different patients. **a** B-mode US in the axial plane demonstrates an enlarged thyroid gland with a coarse and hypoechoic echotexture, exhibiting a “thyroid inferno” pattern at Doppler interrogation (**b**) in a patient with Graves’ disease. **c** Axial B-mode ultrasound of a thyroid gland containing several hypoechoic foci scattered throughout its parenchyma due to lymphocytic infiltration, associated with reduced Doppler flow (**d**), consistent with Hashimoto’s thyroiditis

Hashimoto’s thyroiditis typically shows patchy, hypoechogenicity and a micronodular, “cobblestone” texture, reflecting lymphocytic infiltration and fibrosis (Fig. 6). Gland margins may appear irregular or lobulated, especially in chronic stages, whereas increased vascularity is usually seen in the active inflammatory phase [23]. In contrast, Graves’ disease often presents with homogeneously hypoechoic tissue and marked vascularity on Doppler, described as a “thyroid inferno” (Fig. 6).

Micronodules—ill-defined hypoechoic areas with echogenic rims—are characteristic of Hashimoto’s thyroiditis and correlate with histological lymphocytic infiltration. Fibrotic bands further support this diagnosis. Mild cervical lymphadenopathy can also be present.

Structured reporting systems such as DTD-TIRADS are recommended to improve diagnostic consistency and guide follow-up [24]. Scintigraphy is reserved for hyperthyroid patients to differentiate Graves’ disease from toxic multinodular goitre, with Iodine-123 preferred due to specificity, though technetium-99m is more accessible. On [^18^F]FDG PET-CT, incidental diffuse thyroid uptake has been linked to benign autoimmune activity; however, it is not used for the diagnosis or evaluation of DTD. CT or MRI in DTD is limited to assessing retrosternal goitres and suspected local invasion, with caution advised regarding iodinated contrast if radioiodine therapy is planned [25].

Management of DTD is primarily guided by thyroid function status. In euthyroid or hypothyroid patients, US suffices. Hyperthyroid cases require functional imaging for aetiologic confirmation. Follow-up imaging is unnecessary for stable disease unless clinical progression or suspicious findings arise. Reporting should include standardised gland descriptions, risk categorisation, and clear follow-up guidance [24].

## TC

TC is the most common endocrine malignancy, representing 1.8% of global cancers [26]. Its rising incidence is largely attributed to enhanced imaging and surveillance, without a parallel increase in mortality, underscoring the challenge of overdiagnosis [14]. Approximately 5–10% of TCs, especially papillary thyroid carcinoma (PTC), have a familial component [27]. Women constitute 76% of cases, and the incidence rises with age. Risk factors include radiation exposure to the head and neck, iodine deficiency, and autoimmune thyroiditis [27].

### Histologic and molecular classification

Table 2 summarises the histologic and molecular classification of TC.

**Table 2:** Histologic and molecular classification of TC

Category	Subtypes/characteristics
Differentiated thyroid carcinomas (DTCs)	Papillary thyroid cancer (PTC) Follicular thyroid cancer (FTC) Oncocytic thyroid cancer (OTC)
Medullary thyroid carcinoma (MTC)	Neuroendocrine origin (parafollicular C-cells)
Anaplastic thyroid carcinoma (ATC)	Undifferentiated, extremely aggressive
High-grade follicular cell-derived Non-Anaplastic thyroid carcinoma	Poorly differentiated thyroid carcinoma Differentiated high-grade thyroid carcinoma
Primary thyroid lymphoma	Diffuse large B cell lymphoma—most common histological subtype
Metastases	Arising from primary melanoma, breast, lung, or renal cell carcinoma
Other rare variants	Primary thyroid lymphoma, tumours from stromal, salivary gland, thymic, germ cell, parathyroid, or squamous epithelium

PTC is the most common and usually indolent TC, although aggressive subtypes exist [28]. Oncocytic carcinoma has an intermediate-to-poor prognosis [29]. The BRAF p.V600E mutation occurs in 50–60% of PTCs and is linked to worse outcomes [30]. The 2022 WHO classification supports mutation-specific immunohistochemistry for diagnosis [28]. Staging of TC follows the AJCC/TNM 9th edition, with patients < 55 years classified as Stage I in DTCs [31] while MTC and ATC follow unique staging due to their biology.

#### Imaging modalities

US is the primary diagnostic tool, assessing nodules and lymph nodes for malignancy risk factors like microcalcifications and irregular margins. FNA is the standard sampling method, though core biopsy is preferred for suspected lymphoma or ATC. CT and MRI assist in evaluating locoregional tumour extension in advanced disease. However, iodinated contrast may delay radioactive iodine (RAI) therapy [25]. Radioiodine scintigraphy (I-131) is reserved for post-thyroidectomy detection of residual or metastatic disease. ^18^F-FDG-PET-CT is used in cases with elevated thyroglobulin but negative I-131 scans, and for staging high-grade cancers. ^68^Ga-DOTATATE-PET/CT is preferred for MTC and neuroendocrine tumours due to superior somatostatin receptor targeting [32].

### Histologic subtypes and imaging

PTC is monitored using thyroglobulin (Tg); US is key, while CT and PET-CT are reserved for aggressive variants. Regarding follicular TC, imaging cannot reliably distinguish it from benign adenomas. Diagnostic and surveillance tools include US, I-131, and PET-CT. MTC is detected via calcitonin levels, and familial cases (25%) are often part of MEN2 syndromes [33]. CT, MRI, and Ga-68 PET are vital for staging [34]. ATC is rapidly progressive with a median survival of under six months [35]. Full staging with CT and PET-CT is necessary.

### Prognosis

DTCs generally have excellent outcomes. Key prognostic markers include age, sex, tumour size, Thyroglobulin levels, nodal spread, and BRAF status [30]. MTC prognosis depends on calcitonin, RET mutations, and surgical outcomes [34]. ATC has a dismal prognosis despite aggressive treatment [36].

### Multidisciplinary approach

Effective TC care depends on a multidisciplinary team of radiologists, surgeons, pathologists, endocrinologists, and nuclear medicine specialists for optimal treatment planning.

## Summary statement

**Fig. 7:**
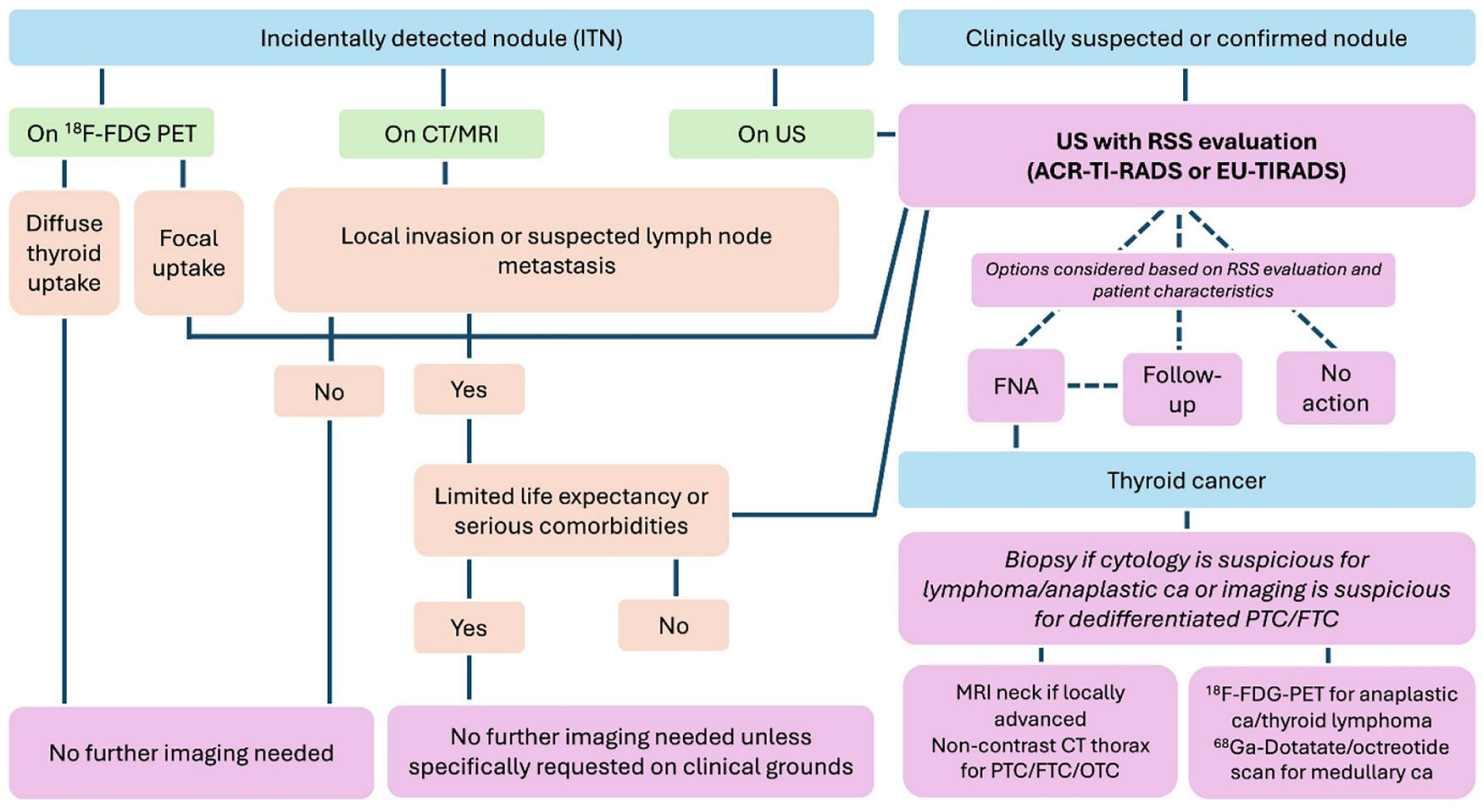
Flowchart of managing ITNs, characterising nodules with US, and imaging TC

Thyroid imaging should be guided by clinical context and risk-based criteria to avoid unnecessary evaluation and overdiagnosis (Fig. 7). Incidental ITNs are common, but routine investigation of nodules without suspicious features (e.g. extrathyroidal extension, cervical lymphadenopathy, or FDG uptake on PET-CT) can lead to avoidable physical, psychological, and financial harms without improving outcomes. Ultrasound-based risk stratification systems (RSS), including ACR TI-RADS and EU-TIRADS, provide evidence-based frameworks that enhance diagnostic accuracy and reduce unnecessary biopsies by incorporating key sonographic features and size-specific FNA thresholds. DTDs, such as Hashimoto thyroiditis and Graves’ disease, are optimally assessed by ultrasound, with characteristic imaging patterns supporting diagnosis in conjunction with thyroid function tests. The rising incidence of TC, largely driven by increased imaging, contrasts with stable mortality rates and highlights the need for evidence-based management. Accurate diagnosis depends on ultrasound, histopathology, and molecular profiling, while optimal care requires multidisciplinary coordination and judicious use of advanced imaging and biopsy techniques.

## Patient summary

ITNs, especially if detected incidentally at imaging, are common and most often harmless. To avoid unnecessary tests and worry, doctors use ultrasound and clinical guidelines to decide when further investigation is needed. Most nodules don’t require treatment unless they show suspicious features. Conditions like Hashimoto’s or Graves’ disease are usually diagnosed with ultrasound and blood tests. Care of patients with TC is guided by a team of specialists to ensure accurate diagnosis and avoid overtreatment, especially as TC has an excellent long-term prognosis.

## Abbreviations

ACR: American College of Radiology
DTC: Differentiated thyroid cancers
FNA: Fine-needle aspiration
ITNs: Thyroid nodules
PTCs: Papillary thyroid carcinomas
RSS: Risk stratification scoring
TC: Thyroid cancer
